# Calcifying Fibrous Pseudotumor of the Pancreas in a Patient With Metastatic Mammary Lobular Carcinoma and Gastric Gastrointestinal Stromal Tumor: A Previously Undescribed Benign Mimic of Metastatic Disease

**DOI:** 10.7759/cureus.9399

**Published:** 2020-07-26

**Authors:** Tony El Jabbour, Nicole Panarelli, Peter Muscarella, Garrison Pease

**Affiliations:** 1 Pathology and Laboratory Medicine, Montefiore Hospital, Bronx, USA; 2 Gastrointestinal and Liver Pathology, Montefiore Hospital, Bronx, USA; 3 Surgery/Pancreatic Surgery, Montefiore Hospital, Bronx, USA; 4 Pathology, Montefiore Hospital, Bronx, USA

**Keywords:** pancreas, calcifying fibrous pseudotumor, solitary fibrous tumor, inflammatory myofibroblastic tumor, gastrointestinal stromal tumor (gist)

## Abstract

Calcifying fibrous pseudotumor, a benign spindle cell tumor, has not been reported previously in the pancreas. Herein, we report a case of pancreatic calcifying fibrous pseudotumor in a 74-year-old female with a history of metastatic breast carcinoma and gastric gastrointestinal stromal tumor (GIST), both confounding the diagnosis and rendering it more challenging. Microscopic examination showed a well-demarcated, paucicellular, densely fibrotic tumor with widespread dystrophic calcifications and sparse, cytologically bland polygonal and spindle cells. Histologic and immunohistochemical work-up helped to exclude relevant differential diagnoses, including metastatic carcinoma, solitary fibrous tumor, inflammatory myofibroblastic tumor, and GIST.

## Introduction

First described as a childhood fibrous tumor with psammoma bodies by Rosenthal and Adbul-Karim, and later designated as calcifying fibrous pseudotumor by Fetsch et al. in 1993, calcifying fibrous pseudotumor is a benign, usually solitary, spindle cell lesion [1-2]. It shows a predilection for the deep soft tissues, body cavities, including the peritoneum or pleura, and subcutaneous locations, such as the head and neck, trunk, and inguinal region [2-4]. These tumors are less common but well-documented in the gastrointestinal tract, including the stomach, esophagus, small intestine, colon, and rectum, and appendix [5-8]. Regarding solid organs of the gastrointestinal tract, two cases have been reported in the liver [8]. Herein, we describe the first case of this tumor in the pancreas. These tumors appear as sharply demarcated, non-encapsulated, oval to round masses with a firm tan cut surface, and occasional yellow calcifications. Histologically, they comprise circumscribed masses of hyalinized collagen with scattered variable psammomatous or dystrophic calcifications and variable lymphoplasmacytic infiltrate [2].

## Case presentation

A 74-year-old woman was referred for the surgical evaluation of a pancreatic body mass. One year prior to presentation, she was diagnosed with invasive lobular carcinoma of the right breast with regional lymph node metastases. It showed strong expression of ER, PR, GATA3, and AE1/AE3 by immunohistochemical (IHC) stains, and was focally positive for mammaglobin and gross cystic disease fluid protein 15 (GCDFP-15). The patient underwent a lumpectomy, chemotherapy, radiation therapy, and was taking an aromatase inhibitor at the time of presentation. 

Magnetic resonance imaging (MRI) demonstrated an incidental large hypervascular mass in the pancreatic body. She denied any abdominal pain or other symptoms. There was no personal or family history of pancreatitis, diabetes, or pancreatic cancer. Serologic studies were performed to evaluate the possibility of a pancreatic neuroendocrine tumor or adenocarcinoma, which revealed an elevated chromogranin at 249 ng/mL (normal: 25 - 140 ng/mL) and a normal cancer antigen (CA)-19-9 at 26 U/mL (normal: < 37 U/mL). Endoscopic ultrasound (EUS) demonstrated an irregular hypoechoic mass in the pancreatic body but yielded a non-diagnostic fine-needle aspiration biopsy (FNAB). A follow-up computed tomography (CT) scan demonstrated a stable 3.2 cm peripherally calcified, hypodense, lobulated pancreatic body lesion without compression of the pancreatic duct. 

**Figure 1:**
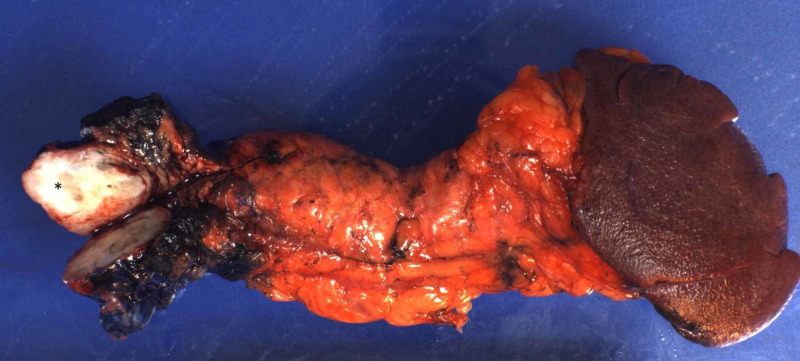
Gross image demonstrating a well-circumscribed tan-white calcified mass of the pancreatic body

For a definitive diagnosis, a distal pancreatectomy with splenectomy was performed. Sectioning of the gross specimen revealed a white to gray, calcified, well-defined 4.0 x 4.0 x 2.5 cm mass confined to the body of the pancreas (Figure 1).

A separate 0.7 cm gastric nodule was also submitted for microscopic examination.

**Figure 2:**
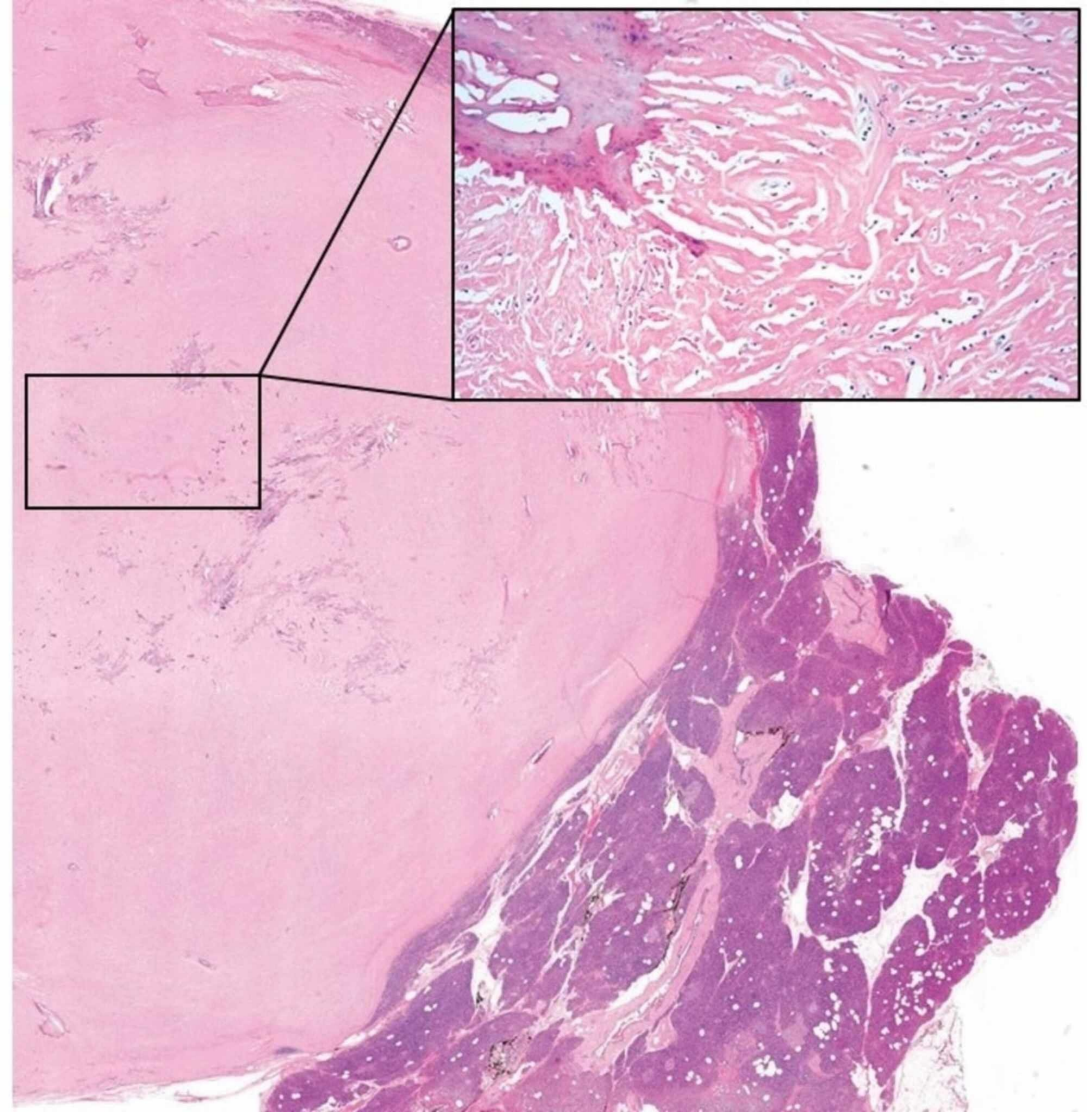
Scanning microscopic view demonstrating a well-demarcated mass from the uninvolved pancreatic parenchyma. Insert: Medium power view showing a paucicellular fibrotic tumor with calcifications.

**Figure 3:**
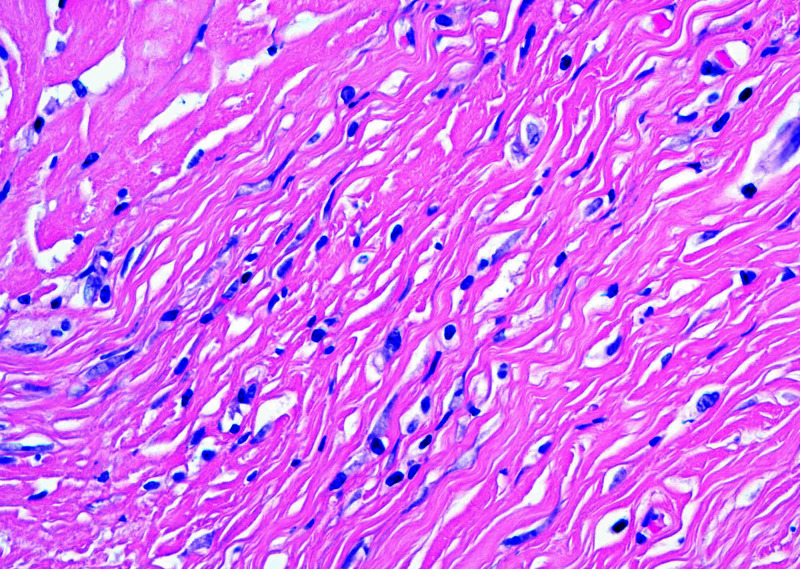
Higher power magnification demonstrating bland polygonal cells embedded within the dense stroma, with focal architecture/pattern potentially mimicking a linear arrangement of carcinoma

**Figure 4:**
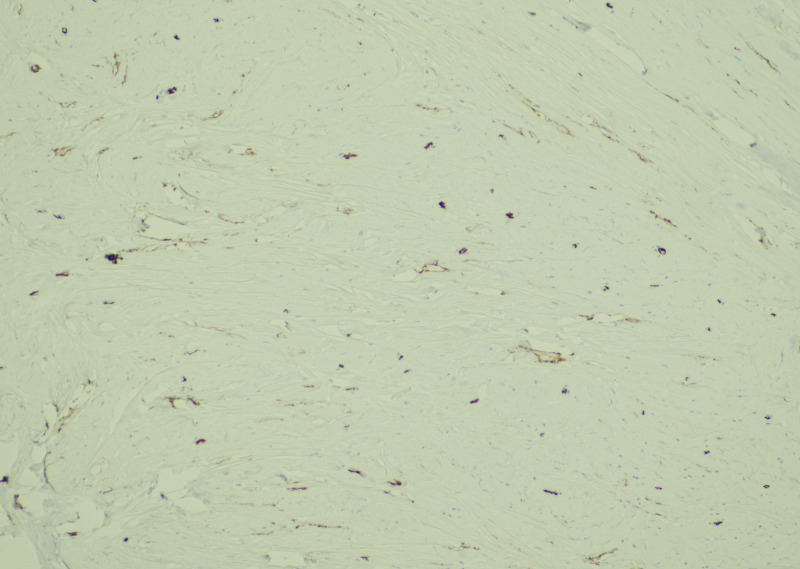
Low-power view of pancytokeratin immunohistochemistry showing diffuse positivity of the polygonal infiltrating cells

Microscopically, the mass was limited to the pancreas and was sharply-demarcated from the surrounding pancreatic parenchyma. It consisted of a paucicellular, densely fibrotic tumor with widespread dystrophic calcifications. Sparse, cytologically bland polygonal and spindle cells were embedded within dense collagenous and fibrotic stroma, focally compressed into arrangements resembling a linear growth pattern. A mild lymphoplasmacytic infiltrate with rare eosinophils was also present (Figures 2, 3). IHC workup revealed expression of AE1/AE3, CAM5.2, SMA, CD34 (patchy), and lack of desmin, ER, PR, HER2, GATA3, GCDFP-15, mammaglobin, CK7, CK20, synaptophysin, chromogranin, CDX2, and S100 expression. Beta-catenin showed membranous, but not nuclear staining of cells within the lesion. A c-KIT stain labeled mast cells but did not stain the lesional spindle cells. An IgG immunostain highlighted intratumoral plasma cells, but only rare cells were labeled with the IgG4 antibody (Figure 4).

Additional workup was performed to evaluate the possibilities of benign and malignant entities. Negative staining for BCL2 and STAT6, the absence of staghorn vasculature, as well as negative staining for ALK1 with sparse inflammatory infiltrate, ruled out a solitary fibrous tumor and inflammatory myofibroblastic tumor, respectively. An elastic stain showed only occasional nodular aggregates of elastic fibers suggestive of obliterated blood vessels, excluding the possibility of a nodular-elastosis-like lesion. Stains for AE1/AE3 and CAM5.2 were favored to be non-specific, especially in light of absent immunoreactivity for CK7, ER, and PR. A final diagnosis of calcifying fibrous pseudotumor involving the pancreas was rendered.

The separately submitted gastric nodule was diagnosed as a GIST, spindle cell type, with no risk of progressive disease on the basis of positive immunohistochemical expression of c-KIT and CD34, and negative S100, SMA, and desmin.

## Discussion

Calcifying fibrous pseudotumor has not been previously described in the pancreas. The differential diagnosis of a well-circumscribed pancreas tumor is broad. In our case, it was important to rule out metastatic carcinoma first and then consider gastrointestinal stromal tumor, solitary fibrous tumor, and inflammatory myofibroblastic tumor [5]. The differential diagnoses of gross and histologic pathology are discussed here. 

In the case of a well-circumscribed tumor in the pancreas, diagnostic considerations should include metastatic disease, especially in a patient with a known history of carcinoma. While primary tumors of the stomach and kidney are the most common that occur as metastasis to the pancreas in surgical specimens, primary tumors of the lung, gastrointestinal tract, kidney, breast, and lymphoma are the most common primary tumors to metastasize to the pancreas in autopsy series [9]. In the current case, the fibrotic and paucicellular bland spindle cell lesion did not resemble carcinoma and was negative for cytokeratins and other IHC markers specific to the breast. 

As the patient had a simultaneous diagnosis of GIST of the stomach, “burnt-out” GIST was considered [6, 10]. Though GIST has rarely occurred as a primary in the pancreas, the negative expression of c-KIT and focal non-specific expression of CD34 did not support this differential diagnosis [11]. 

Solitary fibrous tumor, a collagen-rich spindle cell tumor with alternating hypercellular and hypocellular areas and staghorn-like vessels, was considered. Usually being a visceral or pleural-based tumor, the solitary fibrous tumor has been reported in the pancreas [9]. The majority of cases in the literature were in females at a mean age of 53 (range: 24 to 77) who were asymptomatic or presented with non-specific abdominal symptoms [12]. These tumors, however, do not usually show calcification [13]. The present tumor was uniformly fibrotic, devoid of the usual vascular pattern of a solitary fibrous tumor, and negative for STAT6 and BCL2 IHC markers. 

An inflammatory myofibroblastic tumor was also considered, which has a patternless pattern resembling nodular fasciitis made up of spindled ALK1-positive myofibroblasts; occasional cases have stromal calcification [13]. However, the present tumor had only sparse inflammatory infiltrate and was negative for ALK1, essentially ruling out inflammatory myofibroblastic tumor (IMT). A hyalinized leiomyoma was also ruled out on histomorphology and immunophenotypic grounds. 

Though not typically as well-circumscribed as was seen in this case, IgG4-related sclerosing pancreatitis may produce a dense, fibrotic mass with inflammatory infiltrate. Kuo et al. described splenic angiomatoid nodular transformation associated with disseminated abdominal, gastric, and small intestinal calcifying fibrous tumors, which all demonstrated IgG4-positive plasma cells [14]. Subsequently reported gastric calcifying fibrous pseudotumors have been reported to harbor IgG4-positive plasma cells in some but not all cases [10, 15]. Our IHC studies did not demonstrate substantial IgG4+ cell infiltration. Nevertheless, the reported IgG4+ cases of a calcifying fibrous tumor and a possible association with IgG4-related disease is intriguing.

## Conclusions

Reported herein is a case of a calcifying fibrous pseudotumor, the first known occurrence in the pancreas, in a 74-year-old female with a history of metastatic breast carcinoma. This case was particularly challenging because of the patient’s synchronous benign mesenchymal tumor, GIST of the stomach, and history of lobular breast carcinoma, which is notoriously difficult to diagnose at metastatic sites. These possibilities needed to be excluded prior to considering the possibility of a third, unrelated neoplasm. Furthermore, immunohistochemical and morphologic features did not support the diagnosis of any other benign mesenchymal tumor that is known to occur in the pancreas. This case affirms that the differential diagnosis of circumscribed fibrous pancreatic tumors should include calcifying fibrous pseudotumor, especially when other more common benign mesenchymal lesions have been excluded.

## References

[1] Rosenthal NS, Abdul-Karim FW (1988). Childhood fibrous tumor with psammoma bodies. Clinicopathologic features in two cases. Arch Pathol Lab Med.

[2] Fetsch JF, Montgomery EA, Meis JM (1993). Calcifying fibrous pseudotumor. Am J Surg Pathol.

[3] Marino-Enriquez A, Hornick JL (2019). Spindle Cell Tumors of Adults: Calcifying Fibrous Tumor. Practical Soft Tissue Pathology: A Diagnostic Approach, 2nd edition.

[4] Goldblum JR, Folpe AL, Weiss SW (2014). Enzinger and Weiss's Soft Tissue Tumors, 6th edition. L., Weiss SW: Enzinger and Weiss's Soft Tissue Tumors. 6th ed. Philadelphia, PA: Elsevier Saunders.

[5] Larson BK, Dhall D (2015). Calcifying fibrous tumor of the gastrointestinal tract. Arch Pathol Lab Med.

[6] Agaimy A, Bihl MP, Tornillo L, Wünsch PH, Hartmann A, Michal M (2010). Calcifying fibrous tumor of the stomach: clinicopathologic and molecular study of seven cases with literature review and reappraisal of histogenesis. Am J Surg Pathol.

[7] Pezhouh MK, Rezaei MK, Shabihkhani M, Ghosh A, Belchis D, Montgomery EA, Voltaggio L (2017). Clinicopathologic study of calcifying fibrous tumor of the gastrointestinal tract: a case series. Hum Pathol.

[8] Zhou J, Zhou L, Wu S (2019). Clinicopathologic study of calcifying fibrous tumor emphasizing different anatomical distribution and favorable prognosis. Biomed Res Int.

[9] Klimstra DS, Adsay NV (2015). Tumors of the Pancreas. Odze and Goldblum Surgical Pathology of the GI Tract, Liver, Biliary Tract, and Pancreas, 3rd edition.

[10] George SA, Abdeen S (2015). Gastric calcifying fibrous tumor resembling gastrointestinal stromal tumor: a case report. Iran J Pathol.

[11] Tian YT, Liu H, Shi SS (2014). Malignant extra-gastrointestinal stromal tumor of the pancreas: report of two cases and review of the literature. World J Gastroenterol.

[12] Spasevska L, Janevska V, Janevski V, Noveska B, Zhivadinovik J (2016). Solitary fibrous tumor of the pancreas: a case report and review of the literature. Pril (Makedon Akad Nauk Umet Odd Med Nauki).

[13] Nascimento AF, Ruiz R, Hornick JL, Fletcher CD (2002). Calcifying fibrous 'pseudotumor': clinicopathologic study of 15 cases and analysis of its relationship to inflammatory myofibroblastic tumor. Int J Surg Pathol.

[14] Kuo TT, Chen TC, Lee LY (2009). Sclerosing angiomatoid nodular transformation of the spleen (SANT): clinicopathological study of 10 cases with or without abdominal disseminated calcifying fibrous tumors, and the presence of a significant number of IgG4+ plasma cells. Pathol Int.

[15] Zhang H, Jin Z, Ding S (2015). Gastric calcifying fibrous tumor: a case of suspected immunoglobulin G4-related gastric disease. Saudi J Gastroenterol.

